# Monosodium Urate Crystal-Induced Chondrocyte Death via Autophagic Process

**DOI:** 10.3390/ijms161226164

**Published:** 2015-12-08

**Authors:** Hyun Sook Hwang, Chung Mi Yang, Su Jin Park, Hyun Ah Kim

**Affiliations:** 1 Division of rheumatology, Department of Internal Medicine, Hallym University Sacred Heart Hospital, 896, Pyongchon, Anyang, Kyunggi 431-070, Korea; wazzup@hallym.ac.kr (H.S.H.); cjdalsla@hanmail.net (C.M.Y.); sujin153@naver.com (S.J.P.); 2 Institute for Skeletal Aging, Hallym University, Chunchon 200-702, Korea

**Keywords:** monosodium urate crystals, autophagy, osteoarthritis, chondrocyte, cartilage

## Abstract

Monosodium urate (MSU) crystals, which are highly precipitated in the joint cartilage, increase the production of cartilage-degrading enzymes and pro-inflammatory mediators in cartilage, thereby leading to gouty inflammation and joint damage. In this study, we investigated the effect of MSU crystals on the viability of human articular chondrocytes and the mechanism of MSU crystal-induced chondrocyte death. MSU crystals significantly decreased the viability of primary chondrocytes in a time- and dose-dependent manner. DNA fragmentation was observed in a culture medium of MSU crystal-treated chondrocytes, but not in cell lysates. MSU crystals did not activate caspase-3, a marker of apoptosis, compared with actinomycin D and TNF-α-treated cells. MSU crystals did not directly affect the expression of endoplasmic reticulum (ER) stress markers at the mRNA and protein levels. However, MSU crystals significantly increased the LC3-II level in a time-dependent manner, indicating autophagy activation. Moreover, MSU crystal-induced autophagy and subsequent chondrocyte death were significantly inhibited by 3-methyladenine, a blocker of autophagosomes formation. MSU crystals activated autophagy via inhibition of phosporylation of the Akt/mTOR signaling pathway. These results demonstrate that MSU crystals may cause the death of chondrocytes through the activation of the autophagic process rather than apoptosis or ER stress.

## 1. Introduction

The conversion of uric acid, a product of purine degradation, is mediated by uricase, which is lacking in primates, including humans. An increased level of uric acid in serum (>6.8 mg/dL) leads to the monosodium urate (MSU) crystallization and tissue deposition, resulting in acute arthritis and the formation of tophi [1,2]. MSU crystals deposited in the synovium and cartilage activate immune cells such as monocytes, polymorphonuclear cells, and lymphocytes to induce pro-inflammatory cytokines, including interleukin-6, interleukin-1β, and tumor necrosis factor (TNF)-α, leading to cartilage and bone destruction [2,3,4,5]. Aside from inflammation, MSU crystals have been reported to induce chondrocyte death in previous studies [6,7].

MSU crystals activate p38 mitogen-activated protein kinase through phosphorylation of proline-rich tyrosine kinase 2, focal adhesion kinase, paxillin, and their adaptor proteins, leading to nitric oxide production and matrix metalloproteinase-3 expression [8]. In addition, MSU crystals bind to toll-like receptor-2 (TLR-2), resulting in the formation of signaling complexes involving the myeloid differentiation primary response gene 88 (MyD88), Ras-related C3 botulinum toxin substrate 1, and phosphoinositide 3-kinase (PI3K), consequently inducing nitric oxide generation within chondrocytes [9].

It has been postulated that MSU crystals drive chondrocyte death by hindering the nutrient supply to the chondrocytes and increasing catabolic activity within the cartilage matrix [6]. However, the mechanism to explain such an association between MSU crystal deposition in cartilage and chondrocyte death is not clear. Autophagy is a cellular protective mechanism induced by a wide range of stimuli, such as metabolic stress, oxygen depletion, and endoplasmic reticulum (ER) stress [10,11,12,13]. Mammalian target of rapamycin (mTOR), a negative regulator of autophagy, can form a complex with Raptor (mTORC1) or Rictor (mTORC2). The function or regulatory mechanism of mTOR is dependent upon the formation of a complex containing either Raptor or Rictor. mTOR, activated by the PI3K/Akt pathway, inhibits the process of autophagy and activates protein synthesis and ribosome biogenesis through phosphorylation of S6 kinase and the eIF-4E binding protein [14,15,16]. Several previous reports have shown that microtubule-associated proteins 1A/1B light chains 3 (LC3)-II, an autophagy marker, is up-regulated in the chondrocytes of patients with osteoarthritis (OA) [17,18] and that rapamycin, an inhibitor of mTOR, suppresses glucocorticoid-stimulated chondrocyte death [19]. These findings indicate a relationship among autophagy, cartilage degeneration, and chondrocyte death.

In the present study, we demonstrated the type of cell death mechanisms and signaling pathways that contribute to MSU crystal-induced chondrocyte death and investigated whether MSU crystals induce autophagy-related cell death in human articular chondrocytes.

## 2. Results

### 2.1. MSU (Monosodium Urate) Crystals Reduced the Viability of Articular Chondrocytes

**Figure 1:**
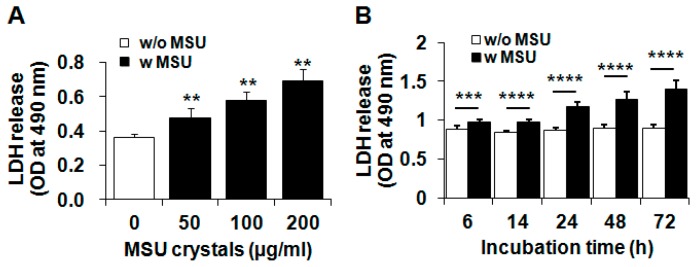
MSU (monosodium urate) crystal-induced chondrocytes death. (**A**,**B**) The effect of MSU crystals on the viability of primary chondrocytes. Primary human chondrocytes were treated (**A**) with various concentrations of MSU crystals (50–200 µg/mL) for 24 h; or (**B**) with MSU crystals (200 µg/mL) for 6, 14, 24, 48, and 72 h. Cell viability was measured with an LDH (lactate dehydrogenase) assay. Data represent the mean ± SD for duplicate experiments from more than three different donors. ** *p* < 0.01, *** *p* < 0.005, **** *p* < 0.001 *vs.* untreated control cells. w/o MSU, without MSU crystals; w MSU, with MSU crystals.

To investigate the effect of MSU crystals on chondrocyte viability, articular chondrocytes were treated with various concentrations (50–200 µg/mL) of MSU crystals for 24 h or with MSU crystals (200 µg/mL) for various time intervals (0–72 h). Lactate dehydrogenase (LDH) release assays showed that MSU crystals significantly reduced the viability of primary chondrocytes from 50 to 200 µg/mL (Figure 1A) and negatively affected the viability of chondrocytes in a time-dependent manner (Figure 1B).

### 2.2. MSU Crystal-Induced Cell Death Is Independent of Apoptosis or ER Stress-Induced Death

**Figure 2:**
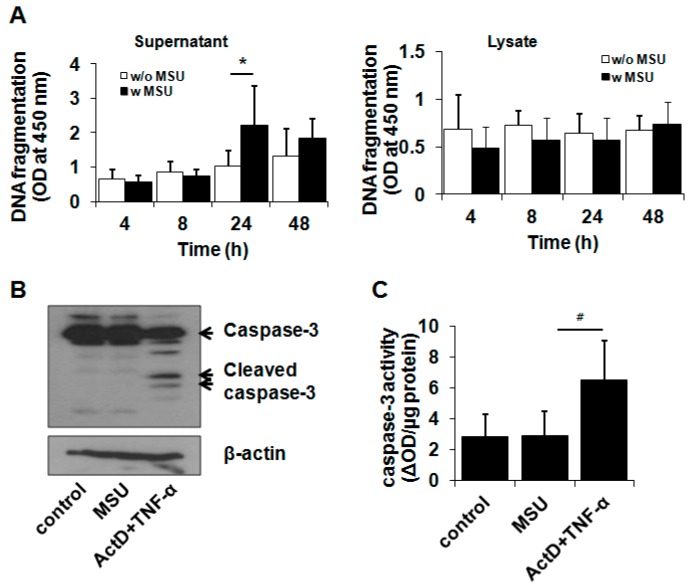
MSU crystal-induced chondrocyte death was not dependent on the apoptosis pathway. (**A**) The effect of MSU crystals on DNA fragmentation in primary human chondrocytes. BrdU-labeled chondrocytes were exposed to MSU crystals (200 µg/mL) for 4, 8, 24, and 48 h. The culture medium and cells were harvested from MSU crystal-treated cells, respectively. Fragmented DNA levels in supernatant and cell lysates were measured using a cellular DNA fragmentation ELISA kit; (**B**,**C**) The effect of MSU crystals on caspase-3 activation in chondrocytes revealed by (**B**) Western blot analysis and (**C**) a caspase-3 assay. Chondrocytes were treated with MSU crystals (200 µg/mL) for 24 h. For apoptosis induction, chondrocytes were treated with Act D (0.2 µg/mL) for 2 h prior to exposure to TNF-α (0.1 µg/mL) for 24 h. (**B**) Cleaved (activated) caspase-3 (17/19 kDa) was measured using Western blot analysis. The Western blot image is representative of three independent experiments from three different donors (*n* = 3); (**C**) Caspase-3 activity in the cell lysates was measured using an ApoAlert caspase colorimetric assay kit. Caspase-3 activity was expressed as optical density changed by caspase-3 per µg protein (ΔOD). Data represent the mean ± SD for duplicate experiments from three different donors (*n* = 3). * *p* < 0.05 *vs.* untreated control cells. ^#^ *p* < 0.05 *vs.* MSU crystal-treated cells.

We examined which type of cell death mechanism was associated with MSU crystal-induced cell death. A DNA fragmentation assay demonstrated that MSU crystals at 200 µg/mL caused DNA fragmentation in chondrocytes at 24 h of incubation (Figure 2A). However, fragmented DNA was significantly found only in the culture medium from MSU crystal-treated cells, not in cell lysates (Figure 2A), suggesting that MSU crystal-induced cell death is related to cell death pathways other than apoptosis. To further confirm whether MSU crystals affect the apoptosis pathway, the activation of caspase-3, an apoptosis marker, was measured with a Western blot and caspase-3 activity assay. Western blot analysis showed that MSU crystals did not induce caspase-3 activation (17 and 19 kDa) compared with actinomycin D (Act D) and TNF-α treatment, which induces chondrocyte apoptosis (Figure 2B). Additionally, consistent with the Western blot analysis, the activity of caspase-3 in MSU crystal-treated chondrocytes was similar to that in untreated control cells, demonstrating that MSU crystals failed to induce apoptosis in chondrocytes (Figure 2C). As expected, Act D- and TNF-α-treated chondrocytes exhibited significantly enhanced caspase-3 activity (Figure 2C). Therefore, our findings demonstrate that MSU crystal-triggered cell death is not related to the apoptosis pathway.

**Figure 3:**
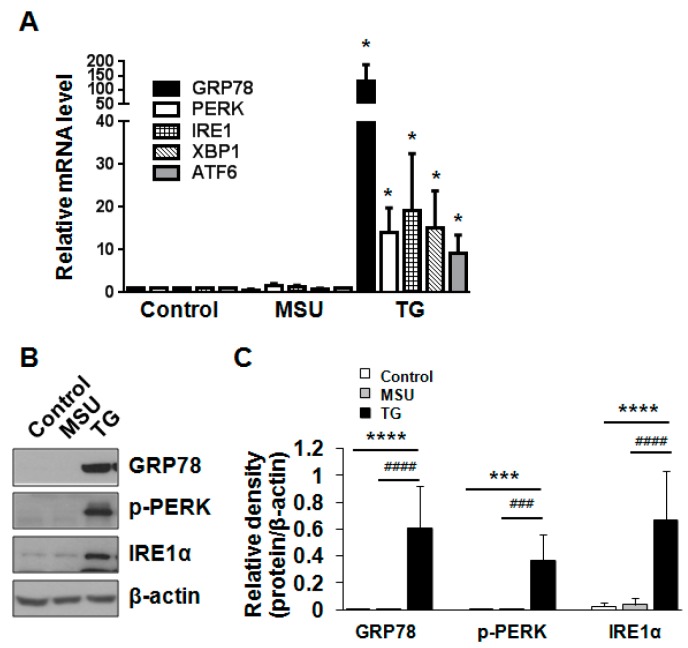
MSU crystal-induced chondrocyte death was not related to ER stress. (**A**) The effect of MSU crystals on the mRNA level of the ER stress markers. Human chondrocytes were incubated with thapsigargin (TG, 0.5 µM) or MSU (200 µg/mL) for 24 h. The mRNA levels of GRP78, PERK, IRE1, XBP1, and ATF6 were measured by RT-qPCR. Data represent the mean ± SD for duplicate experiments from three different donors (*n* = 3). * *p* < 0.05 *vs.* untreated cells; (**B**) The effect of MSU crystals on the protein expression of the ER stress markers. Human chondrocytes were incubated with TG (0.5 µM) or MSU crystals (200 µg/mL) for 24 h. The protein expression of GRP78/Bip, p-PERK, and IRE1α was determined by Western blot analysis. β-actin was used as a loading control. Data are representative of three independent experiments from three different donors (*n* = 3); (**C**) The relative expression level of GRP78/Bip, p-PERK, and IRE1α proteins. Protein density was normalized to β-actin. The bars represent the mean ± SD of triplicate samples from three different donors. *** *p* < 0.005, **** *p* < 0.001 *vs.* untreated control. ^###^ *p* < 0.005, ^####^ *p* < 0.001 *vs.* MSU crystal-treated cells.

Excessive ER stress up-regulates the expression of ER chaperone proteins to increase the folding activity and degrade unfolded or misfolded protein through the ubiquitin system in the cytosol for maintenance of ER homeostasis. In addition, ER stress reportedly induces apoptosis by the transcriptional activation of the C/EBP homologous protein (Chop) [20,21]. We investigated whether MSU crystals decreased cell viability through the induction of ER stress. Chondrocytes were exposed to MSU crystals or thapsigargin (TG), an inducer of ER stress, for 24 h. Real time-quantitative polymerase chain reaction (RT-qPCR) analysis demonstrated that MSU crystals did not alter the mRNA expression of the ER stress markers, including 78-kDa glucose-regulated protein (GRP78)/Bip, protein kinase R-like endoplasmic reticulum kinase (PERK), inositol-requiring enzyme-1 (IRE-1), X-box binding protein-1 (XBP1), and activating transcription factor 6 (ATF6); this differed from TG-treated cells, which up-regulated the mRNA levels of these markers (Figure 3A). In addition, the protein levels of the ER chaperones, including GRP78/Bip, p-PERK, and IRE-1α, were not affected by MSU crystals (Figure 3B,C). However, the treatment of chondrocytes with TG significantly altered the protein expression levels of GRP78/Bip, p-PERK, and IRE-1α compared with MSU crystal-treated cells (Figure 3B,C). Taken together, these results suggest that MSU crystals do not cause chondrocyte death by the ER stress-induced mechanism.

### 2.3. MSU Crystals Induced Articular Chondrocyte Death via Activation of the Autophagy Pathway

**Figure 4:**
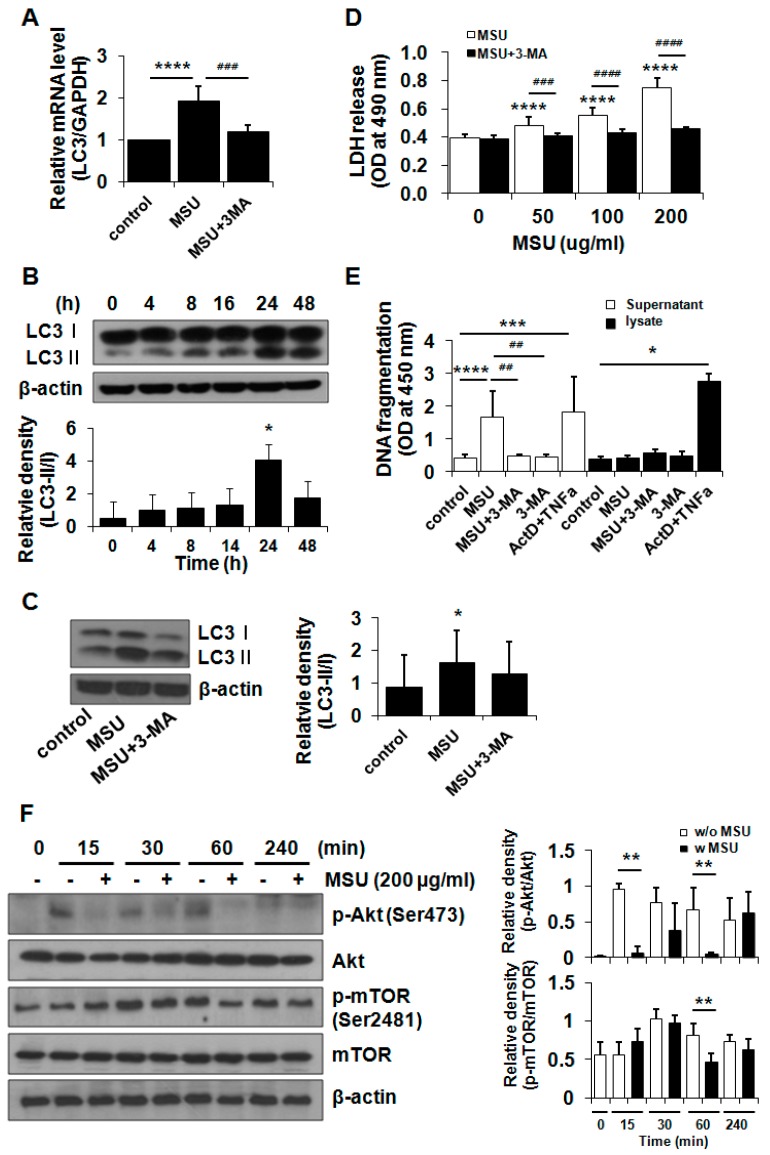
MSU crystal-induced chondrocyte death was mediated by the activation of the autophagy pathway. (**A**) The effect of MSU crystals on the mRNA expression of LC3. Chondrocytes were exposed to 3-MA (2 mM) for 3 h followed by treatment with MSU crystals (200 µg/mL) for 24 h. The level of LC3 was measured using RT-qPCR. Data represent the mean ± SD for duplicate experiments from three different donors (*n* = 3). **** *p* < 0.001 *vs.* untreated cells, ^###^ *p* < 0.005 *vs.* MSU crystal-treated cells; (**B**) The effect of MSU crystals on the conversion of LC3-I or LC3-II, an autophagy marker. Chondrocytes were exposed to MSU crystals (200 µg/mL) for 4, 8, 16, 24, and 48 h. The level of LC3-I to LC3-II was measured using Western blot analysis. Data are representative of three independent experiments from three different donors (*n* = 3). Relative LC3-II/LC3-I ratio in Western blot data. * *p* < 0.05 *vs.* untreated cells; (**C**) MSU crystal-induced autophagy was inhibited by 3-MA. Chondrocytes were exposed to 3-MA (2 mM) for 3 h followed by treatment with MSU crystals (200 µg/mL) for 24 h. The level of LC3-I to LC3-II was measured using Western blot analysis. Relative LC3-II/LC3-I ratio in Western blot data. * *p* < 0.05 *vs.* untreated control cells; (**D**) MSU crystal-induced cell death was suppressed by an autophagy inhibitor. Chondrocytes were exposed to 3-MA (2 mM) for 3 h and incubated with MSU crystals (50, 100, and 200 µg/mL) for 24 h. Cell viability was determined using an LDH release assay. Data represent the mean ± SD for duplicate experiments from three different donors (*n* = 3). **** *p* < 0.001 *vs.* untreated cells. ^###^ *p* < 0.005, ^####^ *p* < 0.001 *vs.* MSU crystal-treated cells; (**E**) The effect of MSU crystals on DNA fragmentation in the presence of 3-MA. Chondrocytes were pretreated with 3-MA (2 mM) for 3 h and incubated with MSU crystals (200 µg/mL) for 24 h. The DNA fragmentation level was measured in the culture medium and cell lysates using a cellular DNA fragmentation ELISA kit. Act D- + TNF-α-treated cells were used as a positive control for DNA fragmentation, an apoptosis marker. Data represent the mean ± SD for duplicate experiments from three different donors (*n* = 3). * *p* < 0.05, *** *p* < 0.05, **** *p* < 0.001 *vs.* untreated cells, ^##^ *p* < 0.01 *vs.* MSU crystal-treated cells; (**F**) Akt/mTOR phosphorylation suppressed by MSU crystals in primary chondrocytes. Chondrocytes were treated with MSU crystals (200 µg/mL) for 0, 15, 30, 60, and 240 min and harvested for Western blot analysis. The levels of Akt/p-Akt (Ser473) and mTOR/p-mTOR (Ser2481) were measured by Western blot analysis. β-actin was used as a loading control. The data are representative of three independent experiments from different donors (*n* = 3). The relative phosphorylation levels of Akt and mTOR proteins. Protein density was normalized to the respective dephosphorylated protein. ** *p* < 0.01 *vs.* MSU-untreated cells. w/o MSU, without MSU crystals; w MSU, with MSU crystals.

During the autophagy process, LC3-I (16 kDa), a cytosolic protein, is changed to LC3-II (14 kDa), a form conjugated to phosphatidylethanolamine of autophagosome, a unique double-membrane structure. We first examined the effect of MSU crystals on the mRNA expression of LC3. RT-qPCR data showed that MSU crystals significantly up-regulated the mRNA level of LC3 but decreased its mRNA level in the presence of 3-methyladenine (3-MA, 2 mM), a blocker of autophagosome formation, by the inhibition of class III PI3K (Figure 4A). To examine whether MSU crystals influence the formation of autophagosome in chondrocytes, the conversion of LC3-I to LC3-II was studied using a Western blot analysis. MSU crystals (200 µg/mL) enhanced the level of LC3-II, which arrived at the maximum level at 24 h of incubation time (Figure 4B). 3-MA significantly suppressed the conversion of LC3-I to LC3-II induced by the MSU crystals (Figure 4C). Furthermore, an LDH release assay demonstrated that pretreatment with 3-MA significantly suppressed MSU crystal-induced cell death (Figure 4D). In line with the LDH release data, DNA fragmentation analysis demonstrated that DNA fragmentation was significantly decreased in the culture medium from MSU crystal- + 3-MA-treated chondrocytes compared with that from MSU crystal only-treated cells (Figure 4E).

We further examined the effect of MSU crystals on the Akt/mTOR/autophagy signaling pathway. Chondrocytes were incubated with MSU crystals (200 µg/mL) for 15, 30, 60, and 240 min, and the activation of Akt/mTOR/autophagy was examined by immunoblot analysis. Notably, the changing of the culture medium at the baseline led to a significant up-regulation of pAkt at 15 min, which was significantly suppressed by the MSU crystals (Figure 4F). mTOR phosphorylation, which was observed from 30 min after the baseline, was down-regulated by the MSU crystals at 60 min (Figure 4F). These results demonstrate that MSU crystals stimulated the autophagy signaling pathway, including the inactivation of Akt and mTOR, thereby leading to decreased chondrocyte viability.

### 2.4. MSU Crystal-Induced Chondrocyte Death Was Independent of Pyroptosis and Necroptosis

**Figure 5:**
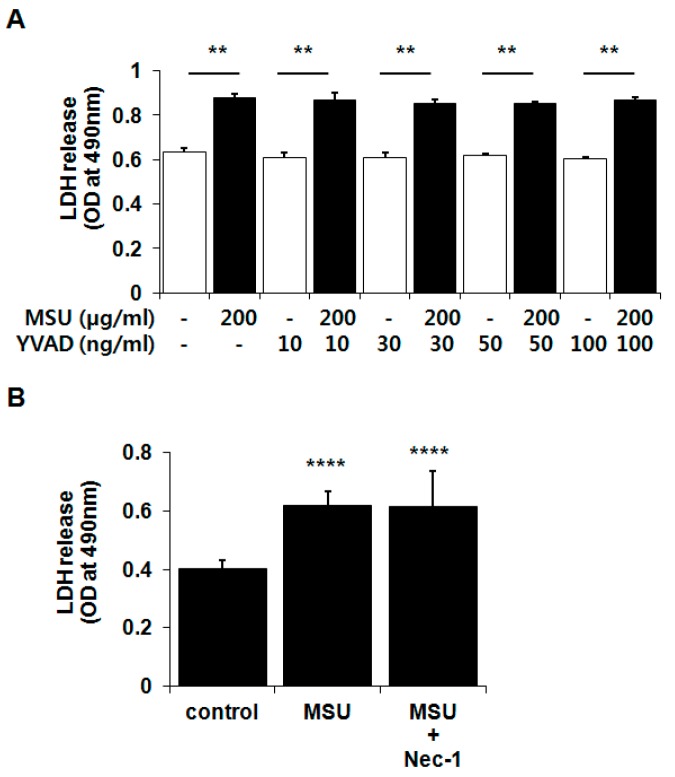
MSU crystal-induced chondrocyte death was independent of (**A**) pyroptosis and (**B**) necroptosis. Chondrocytes were exposed to (**A**) Ac-YVAD-CHO (YVAD; 10, 30, 50, and 100 ng/mL) or (**B**) necrostatin-1 (Nec-1; 30 µM) 3 h prior to treatment with MSU crystals (200 µg/mL) for 24 h. Cell viability was measured by a LDH release assay. Data represent the mean ± SD for triplicate experiments from three different donors. (**A**) ** *p* < 0.01 *vs.* YVAD-only treated cells; (**B**) **** *p* < 0.001 *vs.* untreated cells. (**A**) White bar, without MSU crystals; black bar, with MSU crystals.

Pyroptosis has been recently reported as a novel form of programmed cell death [21,22]. Because pyroptosis is related to caspase-1 activation by the damage-associated molecular pattern-induced inflammasome complex, and excessive activation of caspase-1 is the underlying cause of gouty inflammation, the role of pyroptosis in MSU crystal-induced chondrocyte death was postulated. We also investigated the role of necroptosis, which is induced by ligands of the death receptor family and TLRs and which leads to the formation of necrosomes containing receptor-interacting protein 1 and 3 and caspase-8 [23,24,25]. Chondrocytes were pretreated for 3 h with various concentrations of Ac-YVAD-CHO (YVAD; 10, 30, 50, and 100 ng/mL), a caspase-1 inhibitor, or necrostatin-1 (NEC-1; 30 µM), a necroptosis inhibitor, followed by treatment with MSU crystals (200 µg) for 24 h. Neither inhibitor influenced MSU crystal-induced chondrocyte death (Figure 5A,B). These results demonstrate that MSU crystal-induced chondrocyte death is not mediated by pyroptosis and necroptosis.

## 3. Discussion

**Figure 6:**
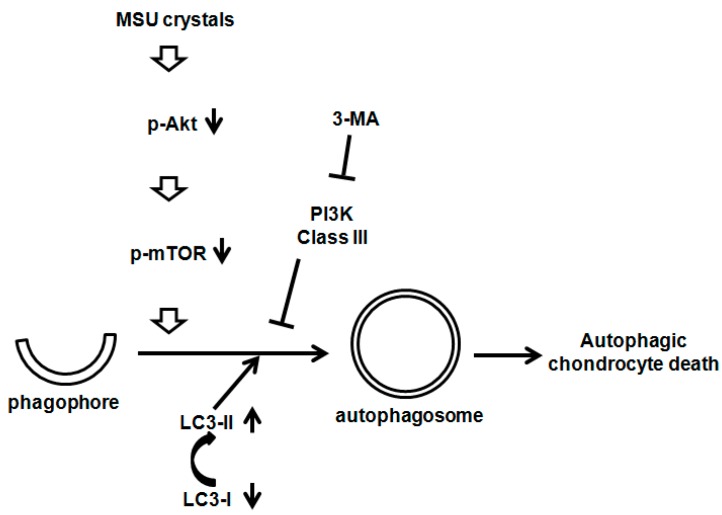
The effect of MSU crystals on the Akt/mTOR/autophagy signaling pathways.

In this study, we demonstrated that MSU crystals cause chondrocytes death, which is neither related to apoptosis nor ER stress, in a dose- and time-dependent manner. MSU crystals up-regulated the mRNA expression of LC3 and activated the autophagic process through the suppression of the Akt/mTOR signaling axis. In addition, a pharmacological inhibitor study provided evidence that the inhibition of class III PI3K by 3-MA, which significantly down-regulates autophagosome formation, subsequently blocks MSU crystal-induced autophagy activation and cell death (Figure 6). Therefore, MSU crystals could induce chondrocyte death via the activation of the autophagic process rather than apoptosis or ER stress.

MSU crystals deposited in the cartilage surface were often observed in the joint tissues of patients with gout [6,26]. Chondrocytes exposed to MSU crystals down-regulated the mRNA expression of cartilage matrix proteins, including type II collagen, aggrecan, and versican, and increased cartilage catabolic enzymes such as matrix metalloproteinases, ADAMTS4, and ADAMTS5, resulting in matrix degradation [6]. In addition, MSU crystals induced nitric oxide production in chondrocytes via TLR-2 signaling and NF-kB, resulting in an overall balance toward cartilage catabolism [9]. While soluble MSU does not affect chondrocyte viability and proteoglycan synthesis, MSU crystals were found to reduce the viability of cartilage matrix-embedded or isolated primary human chondrocytes independently of apoptosis [6].

We examined the MSU crystal-induced chondrocyte death mechanism in further detail. First, the ER stress mechanism was examined because previous studies have demonstrated that it is related to cartilage degradation [27,28,29,30,31,32,33]. ER stress induces expression of ER stress-related genes and the activation of apoptosis in chondrocytes of Chop-knockout mice and human OA cartilage [27,28]. ER stress-induced chondrocytes showed decreased expression of aggrecan and type II collagen but an increased expression of pro-catabolic factors [28,29,34,35,36]. Furthermore, ER stress-induced genes, including XBP1S and IRE1α, were reported to affect ER stress-mediated apoptosis in chondrocytes [37,38]. On the other hand, uric acid induced ER stress in HBZY-1, rat glomerular mesangial cells [39], and hepatocytes [40]. However, our data demonstrated that MSU crystals had no influence on the levels of ER stress markers in human chondrocytes compared with chondrocytes treated with TG, the ER stress inducer. Therefore, although ER stress has emerged as an inducer of cartilage degeneration in OA, MSU crystals might not induce chondrocyte death via ER stress activation.

Autophagic responses are involved in the pathology of diverse diseases, such as diabetes, neurodegenerative disease, and OA [41,42]. Our findings showed that MSU crystals enhanced the level of LC3-II in chondrocytes and that MSU crystals significantly inhibited the phosphorylation of autophagy upstream regulators, Akt and mTOR. Several reports showed that LC3-II, an autophagy marker, was up-regulated in OA chondrocytes and cartilage compared with normal tissues [17,18], particularly the superficial zone and part of the middle zone in a rat OA model [43]. Previous reports have mostly revealed the protective role of autophagy in chondocyte death and cartilage degeneration [19,44,45,46]. Decreased autophagy was found in OA articular cartilage and in an animal OA model [12], and autophagy activation protected chondrocytes from death [44,45]. Progressive loss of stifle cartilage matrix with age was observed in mice, together with decreased autophagy [46]. Furthermore, rapamycin, an inhibitor of mTOR, reduced cartilage degeneration in bovine and human cartilage explants and in an OA mouse model through the activation of LC3 [44,45] and suppressed glucocorticoid-stimulated chondrocyte death [19]. On the other hand, rapamycin-induced autophagy prevented the accumulation of subdiploid cells in young chondrocytes, while it induced cell death by autophagy in OA chondrocytes, suggesting both a cytoprotective and death-promoting role for autophagy in the pathogenesis of OA [47]. Inhibition of autophagy by 3-MA, an inhibitor of PI3K type III, down-regulated TNF-α-induced chondrocyte death through the suppression of both autophagy and autophagy-induced apoptosis [48].

Based on the data from previous reports and our results, we propose that autophagy promotes chondrocyte survival or death depending on donor age, the presence of OA, and the type of autophagy inducer. While the protective role of autophagy was revealed using an aging-related and surgically induced OA mouse model and mechanical injury-applied bovine and normal human cartilages [12,44,45], we utilized chondrocytes obtained from patients with OA. As in gout, the induction of autophagy and cell death in response to MSU crystals is a more rapid process than the process of OA, which may also partly explain the discrepancy.

MSU crystals were reported to increase LC3-II expression and p62 accumulation, a selective autophagy receptor, in murine macrophage [49]. Interestingly, IL-1β upregulation and caspase-1 activation was associated with p62 activation, suggesting the role of autophagy in the regulation of MSU crystal-induced inflammation. Recently, many studies demonstrated that MSU crystals are implicated in the formation of neutrophil extracellular traps (NETs), which are composed of DNA, histones, granular enzymes, and anti-microbial proteins. “NETosis” is a cell death pathway distinguished from other cell death pathways like apoptosis and necroptosis [50,51,52]. The aggregated structures that form in MSU-stimulated neutrophil cultures are similar to tophi, which are the major player of joint destruction in gout [53]. It was thus suggested that aggregation of NET may represent early tophus formation and serve to control acute inflammation by storing MSU crystals [51].

## 4. Experimental Section

### 4.1. Materials

Antibodies to caspase-3, cleaved caspase-3, LC3, phosphorylated-PERK, GRP78/Bip, IRE-1, Akt, p-Akt (Ser473), mTOR, and p-mTOR (Ser2481) were purchased from Cell Signaling Technology (Beverly, MA, USA). Horseradish peroxidase-conjugated secondary antibodies were obtained from Santa Cruz Biotechnology (Santa Cruz, CA, USA).

### 4.2. Sources of Tissues

Cartilage tissues were obtained from patients with OA undergoing total knee replacement surgery who were diagnosed according to the American College of Rheumatology criteria [54]. The collection and use of human samples were reviewed and approved by the institutional review board of Hallym University Sacred Heart Hospital (Anyang, Korea, approval number 2013-I022). All patients provided written informed consent.

### 4.3. Chondrocyte Isolation

Human chondrocytes were isolated as described previously [55]. Briefly, articular cartilage was dissected into small pieces and sequentially digested with protease and collagenase in Dulbecco’s modified Eagle’s medium (DMEM; Life Technologies, Frederick, MD, USA) containing 1% penicillin/streptomycin. Chondrocytes were cultured in DMEM with 10% fetal bovine serum in a humidified atmosphere of 5% CO_2_. First-passage cultured chondrocytes were used for all experiments.

### 4.4. MSU Preparation

MSU (Sigma, St. Louis, MO, USA) was dissolved in boiling water containing 1 N NaOH. After the pH of MSU solution was adjusted to 8.5, the solution was cooled gradually by stirring at room temperature. The crystals were collected by centrifugation at 3000× *g* for 2 min at 4 °C, evaporated, and sterilized by heating at 180 °C for 2 h. Finally, the MSU crystals were suspended at 20 mg/mL in sterile endotoxin-free phosphate buffered saline. Endotoxin contained in the MSU was quantified using a Pierce LAL Chromogenic Endotoxin Quantification Kit (Thermo Fisher Scientific, Waltham, MA, USA). Only endotoxin-free MSU were used in our experiments.

### 4.5. Cell Viability Assay

A LDH release assay was performed to measure cell viability using the CytoTox96 Non-radioactive cytotoxicity assay kit (Promega, Madison, WI, USA). Cells were seeded in wells of a 96-well plate and treated with various concentrations of MSU crystals for indicated times. After incubation, supernatants obtained from each well were transferred to wells on a new plate. The substrate solution was added to each well, and the plate was incubated with gentle shaking for 30 min at room temperature. The optical density was measured at 490 nm using a Thermo scientific Multiskan Go Microplate Spectrophotometer (Thermo Fisher Scientific Inc., Vantaa, Finland).

### 4.6. Colorimetric TUNEL Assay

A TUNEL assay was performed using a cellular DNA fragmentation ELISA kit from Roche Diagnostics (Mannheim, Germany) according to the manufacturer’s instructions. Briefly, cells were labeled with bromodeoxyuridine (BrdU) by incubation in a BrdU-labeling solution for 12 h. Following treatment with the MSU crystals, the culture medium and cell lysates from each sample were transferred into a well on a 96-well flat-bottom microplate precoated with an anti-DNA antibody. After DNA-antibody binding complexes were formed, these complexes were fixed and the DNA was denatured by microwave irradiation. An anti-BrdU-POD conjugate solution was added to each well of the microplate and left overnight at 4 °C. The microplate was incubated with the substrate solution for 1 min by shaking. The optical density was measured at 450 nm within 5 min using a Thermo scientific Multiskan Go Microplate Spectrophotometer.

### 4.7. Caspase-3 Activity Measurement

Caspase-3 activity in the cells was measured using an ApoAlert caspase colorimetric assay kit (Clonetech, Mountain View, CA, USA) according to the manufacturer’s instructions. Briefly, cells were cultured in a 96-well plate at 2 × 10^6^ cells/well and treated with MSU crystals for indicated times. Ten microliters of supernatant from cells that had been lysed with a cell lysis buffer was incubated with a reaction buffer on ice for 30 min, followed by incubation with 50 µM Ac-DEVD-pNA, a caspase-3 substrate, for 1 h. Absorbance at 405 nm was measured using a Thermo scientific Multiskan Go Microplate Spectrophotometer.

### 4.8. RT-qPCR Analysis

Total RNA was extracted using a standard protocol with TRIzol reagent (Invitrogen, Carlsbad, CA, USA). The first-strand cDNA was synthesized from 2 µg total RNA using the Molony murine leukemia virus reverse transcriptase (Promega, Madison, WI, USA). The PCR was performed using a QuantiFast SYBR Green PCR kit (Qiagen, Hilden, Germany) and the StepOnePlus real-time PCR system (Applied Biosystems, Foster, CA, USA). Glyceraldehydes 3-phosphate dehydrogenase (GAPDH) was used as a reference gene. Primer sequences were as follows: LC3 forward 5′-ACC CAG AAG AAG CTG AAC GA-3′, reverse 5′-CTC ATT TGC TGC TTG TTC CA-3′; XBP1 forward 5′-GGA GTT AAG ACA GCG CTT GG-3′, reverse 5′-ACT GGG TCC AAG TTG TCC AG-3′; GRP78 forward 5′-TAG CGT ATG GTG CTG CTG TC-3′, reverse 5′-TTT GTC AGG GGT CTT TCA CC-3′; ATF6 forward 5′-GCC TTT ATT GCT TCC AGC AG-3′, reverse 5′-TGA GAC AGC AAA ACC GTC TG-3′; PERK forward 5′-CTC ACA GGC AAA GGA AGG AG-3′, reverse 5′-AAC AAC TCC AAA GCC ACC AC-3′; IRE1 forward 5′-CGG CCT TTG CAG ATA GTC TC-3′, reverse 5′-CGG CCT TTG CAG ATA GTC TC-3′.

### 4.9. Western Blot Analysis

Treated cells were lysed in a lysis buffer (50 mM sodium acetate, pH 5.8; 10% sodium dodecyl sulfate (SDS); 1 mM ethylenediaminetetraacetic acid; 1 mM phenylmethylsulfonyl fluoride; and 1 µg/mL aprotinin) at 4 °C. β-actin-normalized equal amount of proteins were resolved by 10% SDS-polyacrylamide gel electrophoresis (SDS-PAGE) and transferred to polyvinylidene difluoride membranes (Millipore, Billerica, MS, USA). After blocking with 5% non-fat milk in a TBS-T buffer (25 mM Tris-HCl; 140 mM NaCl; and 0.1% Tween 20, pH 7.5), the membranes were probed with primary antibodies against caspase-3, LC3-I, GRP78, p-PERK, IRE1-α, or β-actin as well as horseradish peroxidase-conjugated secondary antibody. The membrane was developed using an enhanced chemiluminescence kit (Santa Cruz Biotechnology, Santa Cruz, CA, USA).

### 4.10. Statistical Analysis

Results are expressed as means ± standard deviation (SD). Statistical analysis was performed using a Mann–Whitney *U* test or two-way analysis of variance. A value of *p* < 0.05 was taken to indicate statistical significance.

## 5. Conclusions

In conclusion, we demonstrated that MSU crystals significantly reduced the viability of primary articular chondrocytes. Our data using various pharmacological inhibitors or inducers of well-known cell death mechanism show that MSU crystals induced chondrocyte death; this process was dependent on enhanced autophagy signaling but independent of other cell death mechanisms, including apoptosis, ER stress-induced death, necroptosis, and pyroptosis. Therefore, modulation of autophagy signaling could be a critical therapeutic target to protect cartilage damage from gouty inflammation caused by MSU crystals.
